# New Plants, New Resources, New Knowledge: Early Introductions of Exotic Plants to Indigenous Territories in Northwestern North America

**DOI:** 10.3390/plants12173087

**Published:** 2023-08-28

**Authors:** Nancy J. Turner

**Affiliations:** School of Environmental Studies, University of Victoria, Victoria, BC V8W 2Y2, Canada; nturner@uvic.ca

**Keywords:** indigenous food systems, native plant species, northwestern North America, introduced species, changes in food and medicine

## Abstract

Plants have always been important for the Indigenous Peoples of Northwestern North America. Collectively, these peoples named and used hundreds of different native plant species, along with diverse animal species. When traders and settlers from Europe and other parts of the world arrived in the region, they brought many new species of plants with them. Some (e.g., turnips (*Brassica rapa*) and onions (*Allium cepa*)), were from Europe, and some (e.g., potatoes (*Solanum tuberosum*)) were from South America or elsewhere. Other plants, like dandelion, *Taraxacum officinale*, probably arrived unintentionally, as weeds. Examining the ways in which the Indigenous Peoples have incorporated these new species into their lexicons and lifestyles provides insight into processes of acquiring and embracing new products and expanding the cultural knowledge base for human societies in general.

## 1. Introduction

Worldwide, humans have exchanged plant, animal, and fungal species, and have transported them from one place to another, since time immemorial (e.g., [1]). This is the primary way that knowledge about these species spreads across cultural, linguistic, and geographic space [2], including maps of cultural territories and ecological zones of the region. Sometimes, the introduction of species has occurred unintentionally, as with the transmission of some diseases, as well as many weeds and insect pests. Often, however, humans have brought species to new locales with the intention to trade, or new migrants have brought new species to provide food, materials, and medicines that are familiar to them, making life and the acquisition of provisions easier and less risky.

For millennia, the Indigenous Peoples of northwestern North America, connected through intermarriage or other ties of kinship or exchange, have brought plant knowledge and practices, and sometimes the plants themselves, with them to their camps, villages, and other nations throughout the region. It is not surprising, therefore, that when the explorers and traders from Europe, Asia, and elsewhere arrived in northwestern North America beginning in the 1700s, they brought numerous species of plants with them, and that many of these were adopted into the knowledge spheres, cultural practices, and lexicons of the region’s First Peoples.

Here, I consider a broad range of plant introductions to northwestern North America, from the time of the first arrivals of Europeans and other outsiders into the region, starting in the late 1700s to the early 1900s. What kinds of species have been imported, and from where? How have they been incorporated into the lifestyles and languages of the Indigenous Peoples of the region? And how have they, both positively and negatively, influenced the culinary and cultural practices of the region’s First Peoples?

Of course, Europeans and other newcomers to northwestern North America encountered many new and useful plants that they, in turn, adopted for their own use. In some cases, their health and lives depended on the local plants introduced to them by Indigenous residents. These plants included many types of food, such as huckleberries (*Vaccinium membranaceum* and other spp.), saskatoonberries (*Amelanchier alnifolia*), cranberries (*Vaccinium oxycoccos*), and wapato (*Sagittaria latifolia*) [3]. Local medicinal plants, such as cascara (*Frangula purshiana*), false hellebore (*Veratrum viride*), and devil’s-club (*Oplopanax horridus*), were also adopted and used in both original and new ways. A Nlaka’pamux elder, Annie York, recalled how the local Chinese people used the leaves of native wild ginger (*Asarum caudatum*) as a poultice for cuts and sprains, and one of the names she used for this plant was “Chinaman’s medicine” [4]. Some of these Indigenous medicines were eventually incorporated into the North American pharmacopoeia, and some Indigenous people later earned money by harvesting these medicinal products and selling them to the drug industry [5].

Nevertheless, there has been an overwhelming influence, culturally and ecologically, on the environments, languages, and lifestyles of Indigenous Peoples in northwestern North America, caused by the plants and plant products introduced, both intentionally and unintentionally, into the region. They serve as examples of how people tend to enfold new entities and experiences into their languages and cultures, and how they, in turn, can be changed, both positively and negatively, by the new products and experiences. It is these species, therefore, and the context and outcomes of their introduction, that are the focus here.

## 2. Methods

The information provided in this paper is based primarily on a survey of ethnographic, historical, and ethnobiological published sources from northwestern North America and the surrounding areas. Some, including my own authored and co-authored publications, are based on interviews and participatory observations with Indigenous knowledge holders and plant specialists over the past few decades, documenting the names, applications, and relationships with plants that are a key part of people’s cultural knowledge systems, cf. [2,4,6,7,8]. Standard methods in collaborative ethnobotanical documentation—first-hand interviews, workshops, field outings, participant observation, reviews of the existing ethnographic and historical literature, and documents—have all been included in these studies. The ethical codes of the Society of Ethnobiology, the International Society of Ethnobiology, and most recently, the Tri-Council Research Ethics requirements [9], including collaborative research and informed consent, have been followed in this work. A compendium of scientific names, their families, original geographic distributions, and lifeforms, based on POWO URL: https://powo.science.kew.org/ (accessed on 15 August 2023), is provided in Appendix A.

## 3. Background

### 3.1. Indigenous Peoples of Northwestern North America

**Figure 1:**
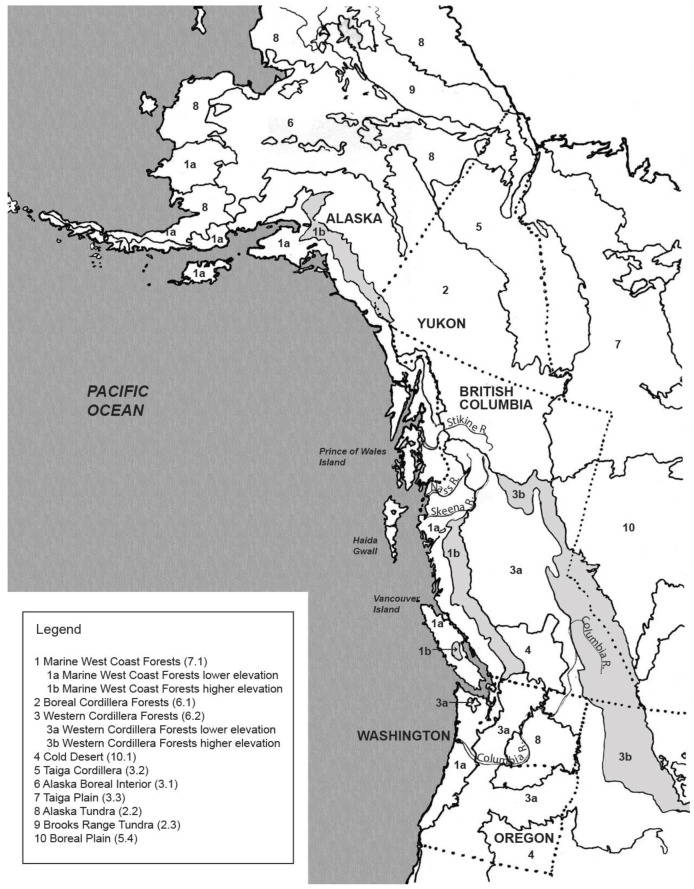
Major vegetation zones of northwestern North America, with accompanying legend. Map drawn by Dr. Nancy Mackin [2] (vol. 1, p. 8).

**Figure 2:**
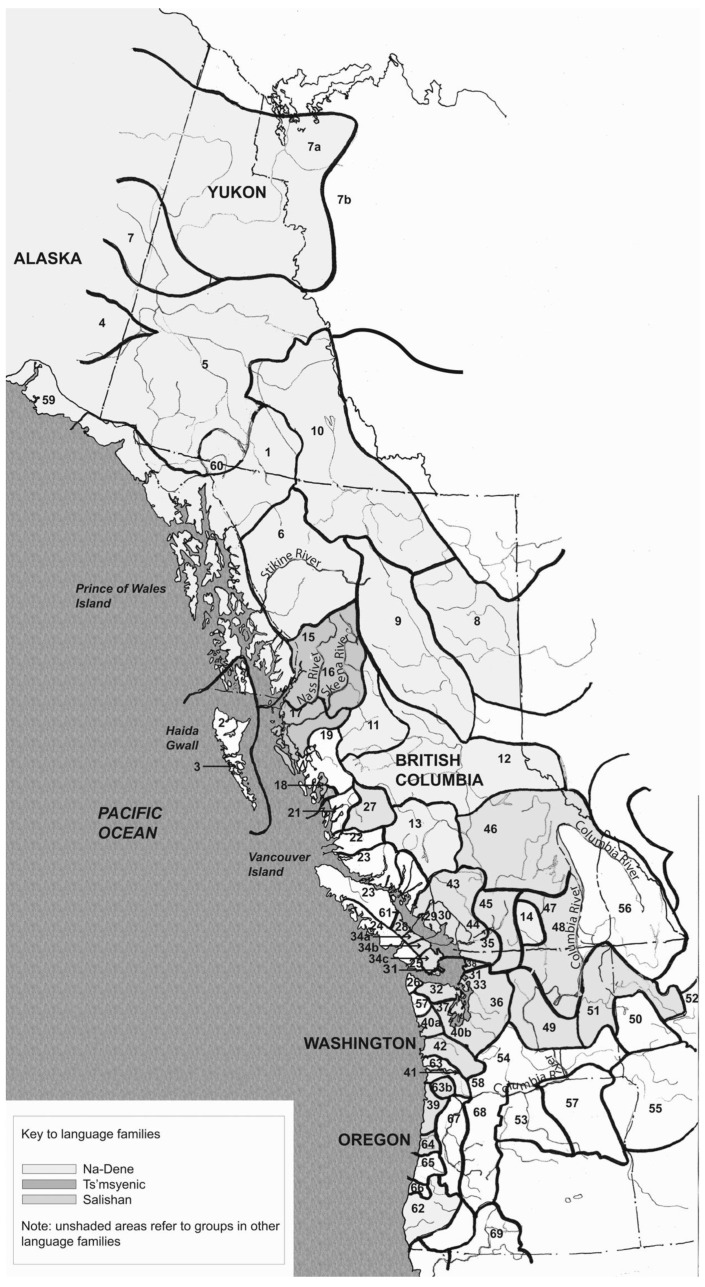
Indigenous languages and cultural areas of northwestern North America; individual groups listed in Appendix B. Map drawn by Dr. Nancy Mackin [2] (vol. 1, p. 11).

Northwestern North America—the region extending from the Columbia River north to central Alaska and east to the Rocky Mountains—includes approximately 20 major vegetation zones, varying in terms of topography, elevation, and latitude [2] (as shown in Figure 1 and Figure 2). Most of these are forested; for instance, there are maritime forests along the west coast at lower and higher elevations, boreal and sub-boreal forests, interior dry forests, interior wet belt forests, and interior high-elevation forests, as well as alpine, tundra, and interior dry grasslands. Each has its own complement of habitats, successional stages, and associated species. In all, there are approximately 2500 native vascular plant species in the region, as well as a diversity of marine algae, fungi, lichens, and bryophytes.

At least from the end of the Pleistocene glacial period, diverse groups of Indigenous Peoples have been living in this part of North America. Altogether, about 50 different languages and major dialects are spoken throughout the region [2]. Several language families are represented, including: Na-Dené (numerous Dene, or Athabaskan, languages, as well as Tlingit and Eyak); Ts’msyenic (Tsimshian); Wakashan; and Salishan; along with linguistic isolates Haida and Ktunaxa. A total of about 270 indigenous plant, algae, and fungi species are named in two or more of these languages. Most of these named species are used as sources of food, materials, and/or medicines, and many have key roles in social and economic relations, stories, and ceremonies.

Even before the first Europeans and their contemporaries from Asia and elsewhere entered northwestern North America, the Indigenous communities were well established as traders. For example, archaeological records of obsidian—the volcanic glass that was treasured since ancient times for arrowheads and cutting implements—have revealed immense trading networks across geographic, linguistic, and cultural boundaries. The source of a particular obsidian object can be identified through “fingerprinting” (e.g., through X-ray fluorescence), so that, for example, obsidian taken from lava flows on Mount Edziza, north of the Skeena River in Tahltan Nation traditional territory, is widespread in the archaeological sites of northwestern British Columbia and southern Alaska, with indications of broad dispersal by different groups of people over thousands of years [10]. Dentalium shells were also widely traded in early times. Originating from the west coast of Vancouver Island, they occur in archaeological sites far inland and even east of the Rockies. Contemporary ethnographers have verified the trading routes for these shells: for example, from coastal peoples to Secwepemc (Shuswap), who occasionally sold them to the Ktunaxa (Kootenai) and the Stoney Nakoda peoples to the south and east [11]. Copper from the terrestrial river drainages of the Wrangell St. Elias Mountain range in Alaska is another widely traded product from ancient times [12].

**Figure 3:**
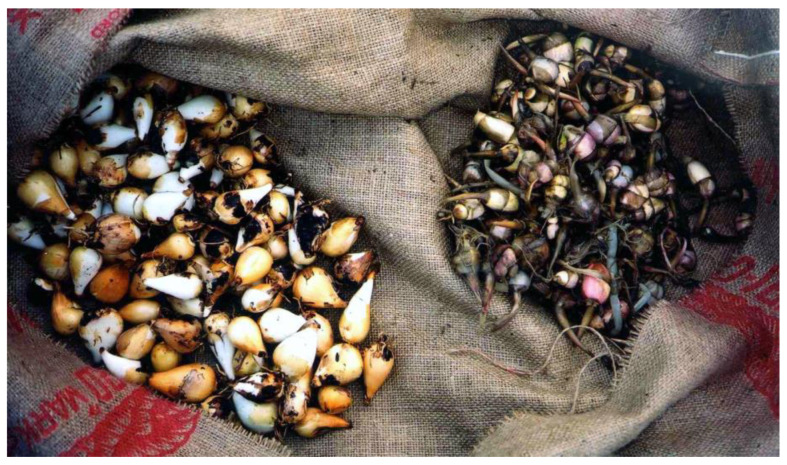
Two prominent original “root vegetables” of northwestern North America harvested from southern Vancouver Island. The original name for *Sagittaria* in some languages was transferred to potato (*Solanum tuberosum*) when it was first introduced (N. Turner, ca. 2000).

**Figure 4:**
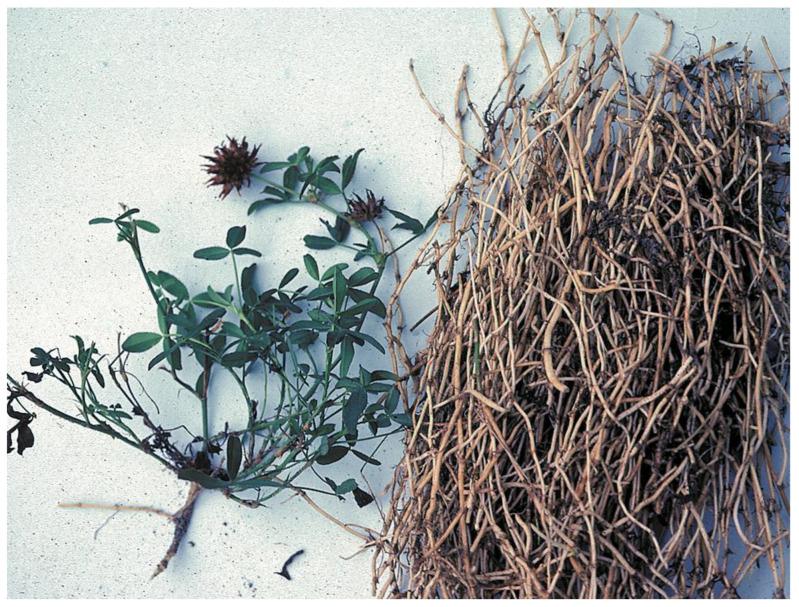
Springbank clover (*Trifolium wormskioldii*) with edible rhizomes from Nitinat Lake, Ditidaht territory, ca. 1988; a key food plant of coastal regions that was traded widely. (N. Turner).

Plants and plant products have been among the goods disseminated widely in the region through trade since time immemorial [2,13]. In some cases, we know that plant distributions have also been extended, with species translocated through human agency. Examples include native tobacco species (*Nicotiana attenuata*, *N. quadrivalvis* var. *quadrivalvis*), manroot (*Marah oregana*), hazelnut (*Corylus cornuta*), blueberry (*Vaccinium myrtilloides*), highbush cranberry (*Viburnum edule*), stinging nettle (*Urtica dioica*), camas (*Camassia quamash*), wapato (*Sagittaria latifolia*) (Figure 3), and springbank clover (*Trifolium wormkioldii*) (Figure 4), to name just a few [14,15].

### 3.2. The New Arrivals from Away

One of the first Europeans to enter the Northwest Coast of North America was British fur trader Captain James Strange. He and his crew arrived at Nootka Sound on the west coast of Vancouver Island in 1786, where they immediately planted a garden of European vegetables. Three years later, Spanish explorer Esté José Martínez and his crew built a garrison at Nootka Sound and also planted a garden, including potatoes, turnips, onions, and cabbage. In 1780, the Spaniards, under Captain Pedro Alberni, established Fort San Miguel in Nootka Sound at Friendly Cove. They planted 19 different grains, legumes, and vegetables and built an irrigation ditch to bring water to their garden [16]. This was the beginning of many new gardens, mostly of European plant foods, that were established in the region. These early gardens were generally associated with forts and trading posts, intended to provide food for the Europeans and other employees living there. However, the gardens, agricultural fields, and livestock pastures the trading companies established also represented the beginning of efforts by European newcomers—traders, settlers, and religious leaders—to gain lands for themselves and to persuade Indigenous occupants to adopt an agricultural lifestyle, including embracing a range of new foods brought in and raised by the traders and colonists.

As more explorers, traders, and settlers arrived, more and more new plants and European-style gardens and pastures were established. For example, in 1811 at Stuart Lake, later known as Fort St James, in the Northeast, North West Company employee Daniel Williams Harmon planted a garden of potatoes, barley, turnips, and other crops—apparently the first to be established on the mainland of what was to become British Columbia. A trading post at Fort Kamloops at the junction of the North and South Thompson Rivers was established in 1812. Fort Langley in the in the Fraser River valley was founded in 1827, and Fort Victoria in 1843. Fort Langley’s farm exceeded 800 hectares and produced potatoes, barley, peas, and wheat, as well as hundreds of pigs and cattle, supplementing the food supplies of many of the Hudson’s Bay Company’s forts as well as its vessels, like the SS *Beaver*, which served to link the coastal communities and forts [17].

These and other forts were major nodes of interaction, bringing together English, Scottish, French Canadian, Hawaiian, Iroquois, Cree, Métis, and local First Nations in the mutual interest of exchange of goods and associated knowledge. The new foods imported and grown in and around the forts—potatoes and other vegetables; fruit trees and berry bushes; and crops of wheat, corn, and other grains, were themselves a major source of trade. As well, intermarriages occurred between the traders and local Indigenous women, who quickly learned about gardening and preparation of the new foods, and the local men were hired to help in food production among other tasks.

Apples, pears, peaches, plums, and cherries; domesticated currants, gooseberries, blackberries, raspberries, and strawberries; as well as rhubarb, turnips, carrots, beans, and peas, were soon being grown in Indigenous peoples’ villages, camps, and gardens and were seamlessly embraced into the language and food systems, often by extensions of the names and applications of their indigenous counterparts. Hay crops were also grown, and the hay used for newly acquired livestock or sold to the traders and settler populations [18].

As well as the new agricultural plants introduced and grown in gardens, fields, and orchards, a number of weedy plants arrived, and some of these were also adopted into cultural use and indigenous vocabularies. Among these, broad-leaved plantain (*Plantago major*) is a prime example. Named “village skunk-cabbage” in Haida, it is associated with frogs in a number of cultures and languages, and is widely known for the application of its leaves as an effective poultice for sores, burns, stings, and infections.

The Cree, Algonkian, and other Indigenous traders and voyageurs accompanying the European newcomers were also evidently responsible for introducing new plants into the region, as well as, in some cases, new uses for plants which were already present [19,20]. For example, Bearberry, or kinnikinnick (*Arctostaphylos uva-ursi*), is native to northwestern North America and is easily available. The berries were eaten, but previously the leaves apparently were not used by western Indigenous Peoples for smoking (The name “kinnikinnick” is said to be an Algonkian term meaning “smoking mixture.” From the fur trade era, the Pacific coastal people learned to use it for smoking, either alone or mixed with tobacco. Similarly, Labrador tea (*Rhododendron groenlandicum*) was used medicinally by Indigenous peoples of the region, but it was only after the fur traders arrived that people started to use it for tea. This is reflected in the names for this shrub in many languages, which incorporate the English word “tea”. For instance, some Haida call it *xàaydaa tiiga* (“Haida-tea”) in the Skidegate dialect, but it is also called k*’usinga xilga* (“tuberculosis-leaves/medicine”) [21]. The knowledge of sweetgrass use (*Anthoxanthum nitens*; syn. *Hierochloë hirta*) by Nlaka’pamux and others may also have originated during the trade era from interactions with Cree, Siksika (Blackfoot), and other peoples from east of the Rockies [22].

Another plant, sweetflag (*Acorus calamus* var. *americanus*), a wetland species well known for its medicinal and spiritual qualities, is widely used by Indigenous People in central and eastern North America [23], but it is rare in British Columbia [24]. Locales where it occurs, such as the mouth of the Salmon River in Secwepemc territory at Salmon Arm, are often associated with Indigenous camps and settlements, suggesting purposeful introduction of the plant, likely by the Cree, Sekani, or other Indigenous traders from eastern or central Canada. It is known to have been transplanted along travel and trade routes in many places [19]. Wild rice (*Zizania aquatica* var. *interior*) also grows in just a few locales in British Columbia (URL https://linnet.geog.ubc.ca/Atlas/Atlas.aspx?sciname=Zizania%20aquatica%20var.%20aquatica&noTransfer=0 (accessed on 15 August 2023)), such as in Stó:lō territory in the Fraser Valley [4], but, again, was evidently imported, likely by Indigenous traders from further east. Chinese and other Asian miners, gardeners, and forestry workers started to arrive into the region around 1860, bringing some of their own foods and adopting some of the food and medicinal plants which they found or learned about from the First Peoples, although there is currently little direct information about these interactions.

Aside from the living plants that were brought into the region by explorers, traders, and settlers, there were also a number of high-profile plant products that were imported early on, and these were soon incorporated into First Peoples’ diets, lifeways, and languages. These included coffee and tea, tobacco, sugar, rice, flour, oatmeal, dried beans, and peas; tropical and subtropical fruits such as bananas, oranges, lemons, cantaloupes, watermelons, and pineapples; as well as dried figs. New materials like *Raphia* palm fibers are used in basketry by Nuu-chah-nulth, along with the newly introduced aniline dyes. Bamboo, which has been used to make knitting needles and other implements, was another new material, sometimes found as driftwood. The new plant products were often named by comparison to known entities. For example, in the Hesquiaht (Nuu-chah-nulth) language, bananas (*Musa x paradisiaca*) were named for their resemblance to slugs, rice (*Oryza sativa*) for its resemblance to maggots, and navy beans (*Phaseolus vulgaris*) after periwinkles (small beach snails). Pineapples (*Ananas comosus*) were named “brushy on the head”, a description of the way the sea mammal hunters wore their hair, gathered up on top of their heads. Dried figs (*Ficus carica*) were named “resembling camas bulbs” in the Ditidaht and Nuu-chah-nulth languages, because of their resemblance to cooked bulbs of *Camassia quamash* in their appearance and sweet taste [8]. Refined white sugar was named “resembling sand” in Hesquiaht, and the Ditidaht name for brown sugar was “alive, alive”, so called because the granules “move around” [8,25]. Commercial tobacco (*Nicotiana tabacum*), in the form of whole leaves and cigars, was imported by the Hudson’s Bay Company and is used universally in almost all language areas for both chewing and smoking. Named after the native species, or after its smoke, this tobacco eventually replaced the Indigenous tobaccos. In addition, it was widely adopted for ceremonial use, sometimes together with kinnikinnick [14,21].

## 4. Results

### 4.1. New Plants, Introduced and Adopted

In the following sections, the plants introduced by the newcomers that were of particular importance to multiple First Nations in northwestern North America are listed within the categories of major usage, starting with root crops and ending with some of the weedy species that gained names and cultural importance within the region’s Indigenous homelands. The information was drawn from published ethnobotanies [4,7,8,21,25,26], with naming information summarized from *Ancient Pathways*, *Ancestral Knowledge* ([2] Supplement 1) (URL https://dspace.library.uvic.ca/handle/1828/5091 (accessed on 15 August 2023)).

#### 4.1.1. “Root” Crops

Starting with the very first gardens established by European and American explorers and traders arriving in the region, root vegetables (including tubers, corms, bulbs, and true roots—all underground storage organs and propagules) have been readily adopted and were major components of the first European-style gardens established by First Peoples there.

Potatoes (*Solanum tuberosum*), in particular, were a prime new food product, readily adopted from the early trading posts and soon being grown throughout the region of northwestern North America, and even widely traded back to the Europeans for other goods [27]. Originating through selective breeding of several wild species many millennia ago, likely in the central Andes of South America, potatoes have been cultivated in countless varieties. They spread northward into central America and were introduced to Europe in the mid sixteenth century, then brought by traders to the Northwest Coast of British Columbia in the late 1700s, although it seems likely that some potato varieties arrived earlier through coastal trade between the Indigenous Peoples along the west coast of the Americas. The closely related varieties known as the “Ozette potato” and “Haida potato” are examples of very early, possibly pre-European introductions [21,27,28,29,30].

**Figure 5:**
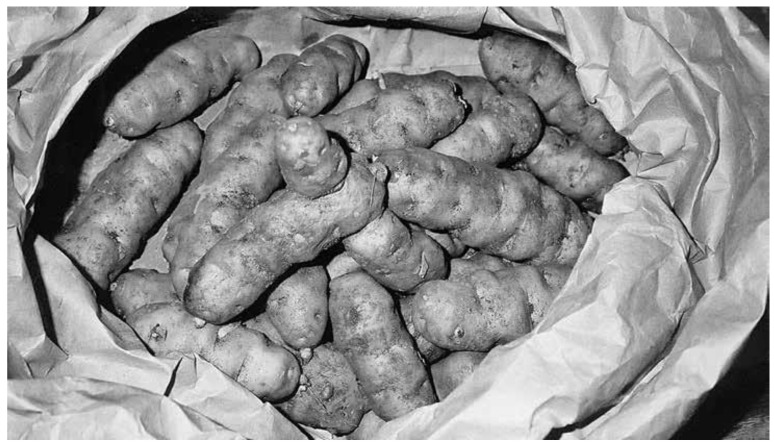
Haida potatoes, grown by Primrose Adams of Masset, Haida Gwaii, and likely from a very early variety, which would have been grown in the trading era. Photo ca. 2004 (N. Turner).

As well, trade between different First Nations groups immediately after Fort Langley was established resulted in potato crops being grown in many Indigenous villages throughout the region even before Europeans had made an appearance in those communities [17,27]. On the north coast at Fort Simpson, near the mouth of the Nass River, naval apprentice John Dunn, who was at the Fort in the 1830s, estimated that some “500 to 800 bushels” of potatoes were brought to the fort by the Haida each year (Figure 5). Within a ten-day period in 1840, he noted, the fort had acquired 1,119 bushels of potatoes from the “Queen Charlotte Islanders”, arriving in “no less than 48 Canoes” [31].

Rumors that some of the coastal names for potato—*sgawsid* (Haida), *sgusí* (Kitasoo), etc.—derived from the English words “good seed” (said to have been conveyed by traders attempting to explain how they should be planted) may be correct. Another explanation, however, is that these terms, with variants common in central and northern coast languages, are derived from Proto-Salish *s-qawts*, for “Indian potato”, including, in some languages, wapato *(Sagittaria latifolia*), and in other cases, possibly Jerusalem artichoke (*Helianthus tuberosus*) [2].

As well as potatoes, other root vegetables were readily adopted and grown in gardens by the region’s First Peoples (see Table 1).

#### 4.1.2. Fruits and Berry Crops

Next to potatoes and turnips, probably the most significant new plants to be introduced to northwestern North America were fruit trees, as well as some domesticated berries. By the 1840s, apple trees and other orchard crops were being introduced. Apples, pears, peaches, plums, and cherries, as well as domesticated currants, gooseberries, blackberries, raspberries, and strawberries, soon took their places in Indigenous peoples’ settlements and gardens and were seamlessly embraced into the languages and food systems, usually by extension of names and applications of their indigenous counterparts. Of these, many have wild relatives native to the region, and because of this, were likely more readily adopted and enfolded into the lifeways and foodways of the First Peoples.

**Figure 6:**
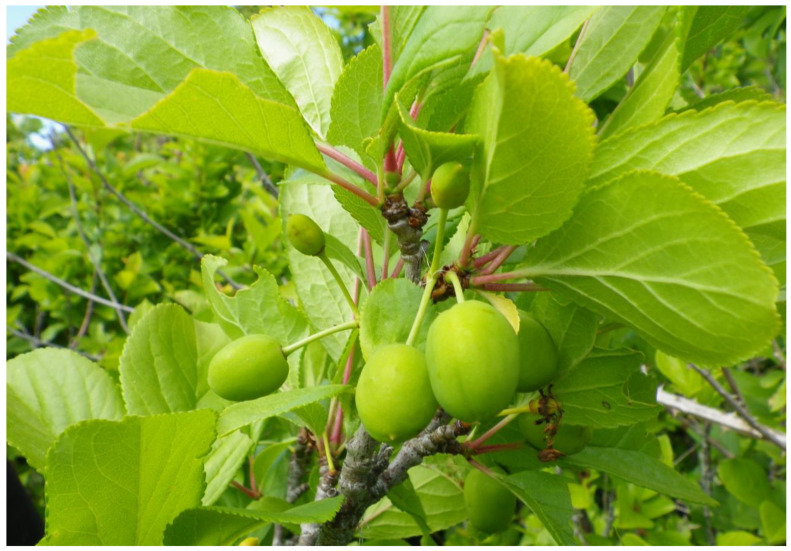
Small unripe plums growing in an Indigenous home site at Tl’ches near Victoria, British Columbia, planted in the 1800s. Photo taken June 2011 (N. Turner).

Even today, fruit trees are found commonly around people’s traditional village sites and camp sites, and in many places are still bearing fruit. Some of these would today be identified as heritage crops, having been replaced in the commercial market by newer varieties. For example, there are apple trees growing at Tl’ches, the islands offshore from Oak Bay in Victoria where Sellemah (Songhees elder Joan Morris) lived as a child [32], identified as a rare variety called “Mother”. This American heirloom apple was first discovered in Massachusetts in the early 1800s, and is rarely seen today (B. Beckwith and R. Duncan, pers comm. 2011). Other rare varieties of cherries, plums, and other fruits (see [33]) (Figure 6) are also encountered in traditional village sites. Some of these new fruits have been incorporated into “forest garden” complexes near settlements, such as in Sts’ailes (Chehalis) territory along the Harrison River, where groups of food, material, and medicine plants have been translocated and maintained [34,35,36,37]. Remnants of the originally introduced blackberry patches can also be found in village sites such as Bella Bella and Hartley Bay. In Bella Coola, Margaret Siwallace was growing particularly large, juicy raspberries which she had obtained originally from a nearby ranch, Cresswell Ranch [38].

The introduced fruits, as well as being enjoyed fresh, would have been prepared for winter storage and trade by cooking them slightly then drying them in the sun, or, in the case of apples, simply storing raw until needed, and then processed. However, canning in jars or cans; making into jams and jellies; and, later, freezing these fruits would have been readily adopted as these technologies became available. Table 2 lists the fruit species that were widely introduced into the region in colonial times to be adopted and grown by First Peoples.

**Table 2:** Fruit and berry crops introduced to Northwestern North America by European and other newcomers in colonial times and adopted as food by Indigenous Peoples.

Introduced Fruit and Berry Species	Notes
*Fragaria X—*(garden strawberry)	A favorite fruit; named after native strawberries (*F. chiloensis*, *F. virginiana*, *F. vesca*); widely grown in gardens; named in over 45 languages
*Malus domestica* (apples, many varieties)	Named after English “apples”, or sometimes after native crabapples (*Malus fusca*); widely planted at village sites; some early varieties still remain; named in over 20 languages
*Prunus avium* (sweet cherry) and *P. cerasus* (sour cherry)	Mostly named after English “cherries”, (e.g., Squamish: “*chi-lis*”); in some cases, after native choke cherry (*Prunus virginiana*); widely planted at village sites; named in at least 8 languages
*Prunus domestica* (plums)	Named after English “plums” or after their big seeds; widely planted at village sites; named in over 10 languages
*Prunus persica* (peaches)	Named after the English name, after their fuzzy texture, or after native fruit; named in at least 5 languages
*Pyrus communis* (pears)	Named after English “pears”, or for their narrowing shape; widely planted at village sites; named in at least 5 languages
*Ribes nigrum* (black garden currant)	Often named after native currant relatives (e.g., *R. hudsonianum*); in some cases after the English name “currants”; widely planted at village sites; named in at least 8 languages
*Ribes rubrum* (red garden currant)	Often named after native currant relatives (e.g., *R. triste*), other red berries like red huckleberries (*Vaccinium parvifolium*) or soapberries (*Shepherdia canadensis*),or in some cases after the English name; widely planted at village sites; named in at least 10 languages
*Rubus armeniacus* (Himalayan blackberry); *R. nemoralis* (cutleaf blackberry), *R. allegheniensis* (Allegheny blackberry), and various domesticated forms	Named after their sharp prickles, rope-like growth form, or after native relatives like trailing blackberry (*R. ursinus*), blackcap (*R. leucodermis*), and salmonberry (*R. spectabilis*); still found growing around many village sites (*R. armeniacus* is very invasive); named in at least 15 languages (Figure 7)
*Rubus* hybrids (loganberry, boysenberry, and related hybrids of blackberries and raspberries)	Introduced sporadically and named after blackcaps (*R. leucodermis*), blackberries (*Rubus* spp.), or native raspberries (*R. idaeus*); named in a few languages (e.g., Hesquiaht Nuu-chah-nulth: “blackcaps belonging to the white-man”, for loganberry)
*Rubus idaeus* (raspberry)	Widely introduced and very popular to grow, especially along the coast where native raspberries do not occur; generally named after wild relatives such as salmonberries (*R. spectabilis*), blackberries (*R.* ursinus), or thimbleberries (*R. parviflorus*); where wild raspberries grow, the garden raspberries are given the same name; wineberry (*R. phoenicolasius*) was also introduced locally to the Stó:lō of the Fraser Valley
Tomato (*Solanum lycopersicum*)	Introduced to Syilx and others in the southern Interior; named after the English name, for their color, or their resemblance to objects (e.g., rose hips); named in at least 5 languages
*Vaccinium corymbosum* (highbush blueberry) and other cultivated blueberry species and varieties	Readily adopted as high-producing relatives of native *Vaccinium* species, especially in the Fraser Valley area; usually named after their wild relatives; named in at least 7 languages
*Vitis vinifera* (grapes)	Introduced in warmer climate areas of the region; more widely known in the form of raisins; named after their English name, sometimes named after wild berries like saskatoons (*Amelanchier alnifolia*), or after the “bunch” forming fruit; raisins named after flies in Hesquiaht; grapes named in at least 7 languages

**Figure 7:**
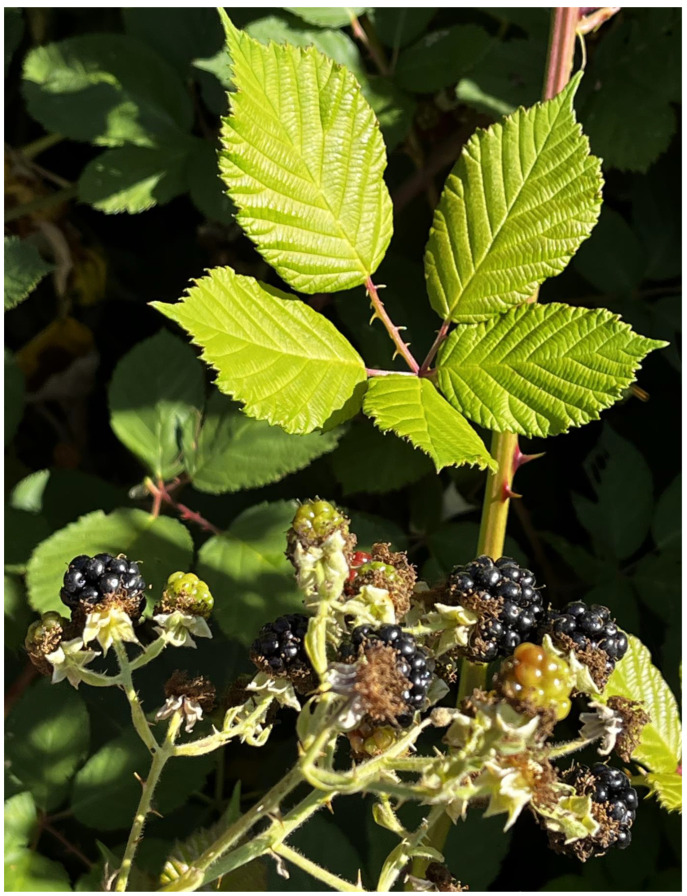
Himalayan blackberry (*Rubus armeniacus*), originally introduced to northwestern North America as a food crop, but soon becoming a weedy invasive species (N. Turner).

#### 4.1.3. Green Vegetables, Legumes, and Squashes

Following the establishment of vegetable gardens at Nootka Sound [16] and at subsequent fur trading posts throughout the region, many vegetable species were imported, readily adopted, and grown in the gardens of First Peoples. These new crops were often compared in terms of appearance, flavor, or use to edible native vegetables and other plants, and were named after these original foods. Many of the new vegetables were seamlessly incorporated into peoples’ diets, to be eaten fresh; cooked in soups and stews; or, in some cases, baked (see Table 3). Five of the vegetable crops originally planted in the Spanish gardens at Nootka Sound [16]—globe artichoke (*Cynara cardunculus*), eggplant (*Solanum melongena*), chickpea (*Cicer arietinum*), green pepper (*Capsicum annuum*), and European angelica (*Angelica archangelica*)—were apparently not readily adopted or were not available for adoption by First Peoples in the region in the early days and are not often mentioned in the accounts or vocabularies of First Nations.

#### 4.1.4. Beverage and Flavoring Plants

Along with the various fruits and vegetables, a few plants were introduced in other capacities, as aromatic flavorings for teas and beverages, for example (Table 4). The introduced varieties of mint (*Mentha* spp.) can still be found growing in First Nations traditional village sites. Hops (*Humulus lupulus*) have had particular importance for generations of Indigenous Peoples of the lower mainland of British Columbia and Vancouver Island, from the late 1800s into the mid 1900s, because many people travelled to hop farms in the Fraser Valley and elsewhere to pick hops for the beer industry. Hops are still grown as decorative vines in some Indigenous communities. As well as gaining knowledge of the hops themselves, people learned from each other at such meeting places, trading goods such as “American grass” (*Xerophyllum tenax*) for basketry and soapberries (*Shepherdia canadensis*), as well as learning about new medicines from their indigenous neighbors.

#### 4.1.5. Grains, Grasses, and Hay Crops, and Introduced Weedy Plants

Along with the new plant species that the First Peoples adopted and grew in their gardens, many also acquired cattle, horses, and other livestock, so the importation, use, and knowledge of hay crops and grains became particularly important. For some, such as Nlaka’pamux and Stl’atl’imx Interior Salish, a semantic shift in vocabulary occurred as people started incorporating these new species—both those which were intentionally cultivated and those that established themselves as weeds. In these languages, the original term for “grass” (particularly the widespread perennial bunchgrass *Pseudoroegneria spicata*) expanded to include “hay”, which was intentionally grown as livestock feed. Similarly, a term previously applied to any low herbaceous plant growth shifted in its meaning towards “weed”, with the implication of ubiquitousness and undesirability of such plants [18]. Table 5 and Table 6 list some of the new grains, grasses, and hay crops that Indigenous Peoples adopted, especially in their new role as keepers of livestock, as well as diverse species of weedy plants. Some of the latter gained new roles as significant sources of food and/or medicine.

**Table 5:** Grasses, grains, and hay crops (see [18]).

Introduced Grasses, Grains, and Hay Crops	
*Avena sativa* (oats)	Adopted and grown in agricultural fields by some first nations; named after English or French names; rolled oats named after cow-parsnip (*Heracleum maximum*) seeds in Haida; this grain named in at least 7 languages
*Hordeum vulgare* (barley)	Planted at Nootka Sound by Spaniards [16]; grown by some First Nations; named in at least 5 languages
*Medicago sativa* (alfalfa)	Adopted and grown as a hay crop by interior First Peoples; now (along with sweet-clovers and timothy) called “real hay” by some (e.g., Syilx/Okanagan)
*Melilotus officinalis* (yellow sweet-clover), *M. albus* (white sweet-clover)	Adopted and grown as hay crops by interior First Peoples; called “real hay” by some (e.g., Syilx/Okanagan)
*Phalaris arundinacea* (reed canary grass)	Introduced early on as a hay crop (although it is possible there was a native subspecies); now a widespread weedy wetland plant; adopted by many basket makers; young stalks are a major material for cedar root basket imbrication; called “Chilliwack grass” by one Nlaka’pamux woman; named in several languages
*Phleum pratense* (timothy grass)	Known as a hay crop; named after wild hay grasses by some; named in at least 5 languages
*Triticum aestivum* (wheat)	Introduced and grown early around trading forts; widely known as the source of flour; adopted as a grain crop by some First Peoples; whole grains are boiled as food by some; named in at least 6 languages (names for flour in virtually all languages)
*Zea mays* (maize, corn)	Originally from Mexico and/or eastern N America; adopted and grown as a grain crop by various First Nations in northwestern N America; named after the French name or resemblance to certain objects (e.g., Nuu-chah-nulth for “salmon eggs”; “tooth” in some Salishan languages); named in at least 15 languages
*Zizania aquatica* var. *interior* (wild-rice)	Introduced early from eastern Canada; likely imported by Cree or other Indigenous traders; grown in the Fraser Valley by Stó:lō

**Table 6:** Weedy plant species (including some used as food or medicinal) introduced by newcomers and named and/or used by Indigenous Peoples of Northwestern North America (see [39]).

Introduced Weedy Plants Named and/or Used	
*Arctium minus* (burdock)	Said to have been introduced with cattle; named for its prickly, sticky burrs (e.g., called “sea urchin” in Nuxalk, and “it sticks to you ground-growth” in Nlaka’pamux); named in at least 13 languages
*Chenopodium album* (lambsquarters)	Introduced as a weed; named in several languages, mostly for its greens; formerly cooked and eaten as a green vegetable; seeds of native *Chenopodium* common in interior archaeological sites; named in at least 9 languages
*Cirsium arvense* (Canada thistle), *C. vulgare* (Scottish thistle)	Weedy species used to bring luck and protection like other prickly plants in some cultures (e.g., WSÁNEĆ); named after native thistles for their sharp spines or prickles; named in at least 25 languages
*Convolvulus arvensis* (field bindweed)	An introduced weed named for its trailing habit; used as a packstrap material by Syilx/Okanagan
*Elymus repens* (quackgrass)	Recognized as a weed that takes over traditional root harvesting areas in interior localities (e.g., Secwepemc)
*Galium aparine* (bedstraw)	Recognized and named for its sticky, burred fruits and its relationship to some native species; considered a plant to be avoided as it might cause the death of loved ones (Syilx/Okanagan)
*Koenigia polystachya* (syn. *Persicaria wallichii*) (Himalayan knotweed) and *Reynoutria japonica* (Japanese knotweed)	Formerly young shoots were probably eaten; imported to a number of reserves (e.g., Hartley Bay, for Himalayan knotweed); brought in by elders as ornamental flower and edible green ca. 1920s and 1930s
*Matricaria discoidea* (pineappleweed)	Known for its scent and as a beverage plant by some; little tops are eaten; used as spiritual medicine and “love medicine”; named in at least 12 languages
*Nasturtium officinale* (watercress)	Introduced by miners and prospectors as an edible green and used by some interior First Nations; named after its aquatic habitat; named in at least 3 languages
*Plantago major* (broad-leaved plantain)	A major weedy plant; long known to Indigenous Peoples of the region; grows commonly in village sites (called “village skunk-cabbage” in Haida); named after frogs in a number of languages; widely used as a medicine for sores, cuts, burns, and stings; named in over 20 languages (Figure 8)
*Ranunculus acris* (meadow buttercup) and other introduced *Ranunculus* spp.	Called “doctor leaves/medicine” (e.g., “*daktaa*” *xilGa*) by Haida; named for its yellow flowers by some; used medicinally by the Haida and others to induce blistering for treating underlying pain; named in at least 12 languages
*Rumex acetosella* (sourgrass or sheep sorrel)	Grows widely as a weedy plant; leaves chewed for their sour flavor, especially by children; generally named for their sour taste (e.g., Nlaka’pamux: *ts’ol’ts’əl’t tək stuyt-úym’x^w^* “sour ground-growth”); named in at least 7 languages
*Rumex crispus* and other *Rumex* spp. (curly dock, and related dock species)	Called by same name as native western dock (*Rumex occidentalis*) in some languages; Saanich name is “coffee grounds plant”; used as medicine by some; named in several languages
*Taraxacum officinale* (common dandelion)	Widely known; leaves sometimes eaten; latex used by some as medicine to remove warts; variously named after English name, color, parachuted fruits, or white latex; named in at least 18 languages
*Tragopogon pratensis* (salsify or goatsbeard)	Recognized as a weed and named using the general term for grasses and grass-like plants (Syilx/Okanagan)
*Trifolium pratense* and other introduced *Trifolium* spp. (red clover and white flowered clovers)	Widely recognized and named variously after their colors, after native clover species, or as “hay” (along with timothy grass, alfalfa, and sweet-clover); replaced native *T. wormskioldii* in many places, but is not generally eaten; named in at least 6 languages
*Verbascum thapsus* (common mullein)	Known as a weedy plant of sagebrush areas in the interior; leaves smoked by some and used medicinally by others (e.g., for tuberculosis [40]), which was possibly learned from European immigrants; called “train’s seeds” in Selish because it was first observed along railroad tracks; named in at least three languages

**Figure 8:**
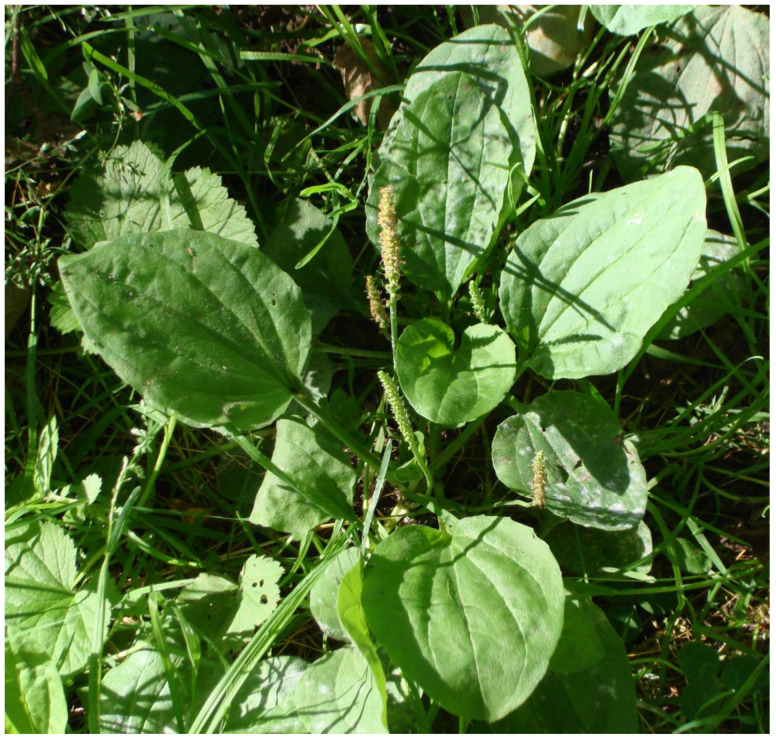
Broad-leaved plantain (*Plantago major*), an introduced plant readily named and used by Indigenous Peoples of northwestern North America as a medicinal poultice for cuts, wounds, bites, and stings (N. Turner).

### 4.2. Introduced Flowers and Ornamental Plants

Along with vegetable gardens and orchards, Indigenous Peoples also enjoyed the new species of flowers and ornamental plants that were brought in by settlers from different parts of the world. In the 1920s or 1930s, for example, the Gitga’at elders of Hartley Bay brought home rhizomes of yellow iris (*Iris pseudacorus*), said to have been obtained from Japanese fishermen in the town of Prince Rupert. The irises spread to a number of gardens and are still growing there in Hartley Bay to the present. Many other garden flowers and ornamental shrubs were introduced to gardens in Indigenous communities, including but not limited to: bachelor’s button (*Centaurea cyanus*), lilac (*Syringa vulgaris*), pansy (*Viola tricolor* and hybrids), and ornamental roses (*Rosa* spp.). These species have names in a number of Indigenous languages which pertain to their color, their scent, or their resemblance to wild relatives.

## 5. Discussion

### 5.1. Incorporating New Species into Languages

The diverse ways in which people have developed and created names for new species they encounter give clues about how languages expand and change over time, as some entities lose salience and others gain prominence in people’s lives [41]. The names of some plants are borrowed outright from the original languages, sometimes adjusted to conform to the sound systems which are more familiar in the adopting languages. Thus, the Lushootseed name for onions, *ʔajədz*, is borrowed from English, and their name for peas, *lipwá*, is borrowed from French. In Dakelh/Carrier (Stuart/Trembleur Lake dialect) the name for oats—*ʔoots*—is borrowed from English, as is the Upriver Halkomelem name, *óts*. Similarly, the Syilx/Okanagan name for cabbage is *kapíts*, from English “cabbage”, and the Tsilhqot’in name for turnips, *danapes*, is likewise a rendering of the English name.

Sometimes, existing terms in an Indigenous language are expanded in their reference to incorporate new but similar entities. When this happens, the original term as it pertains to the original species may have a qualifier or descriptor added to its name: “the *original* X” and/or the new plant or other lifeform may have a qualifier, such as “whiteman’s X” or “English X”. For example, in the case of rhubarb (*Rheum rhabarbarum*), when it was introduced to the gardens of a number of Indigenous groups in the region, its similarity to western dock (*Rumex occidentalis*) was noted by some, and to cow-parsnip (*Heracelum maximum*) by others. Consequently, it was named after one of these two native species in a number of languages. Conversely, in some cases, the wild counterparts came to be known as wild-growing types of rhubarb. In the Masset dialect of Haida, for example, western dock is called *xàadas tł’aaq’ujaa* (literally “Haida rhubarb”) and domesticated rhubarb is called *tł’aaq’ujaa*. In the Skidegate dialect, the name *tl’aangk’uus* is used for both western dock and garden rhubarb.

Similar expansions or transfers of existing names for native species to encompass new and increasingly salient species are very common among the plants noted here: “potatoes” (*Solanum tuberosum*), named after wapato (*Sagittaria latifolia*); currants (*Ribes nigrum*), named after native black currants (*R. hudsonianum*); strawberries (*Fragaria X hybrids*), named after their wild counterparts; introduced thistles (*Cirsium vulgare*, *C. arvense*), after native thistles (*Cirsium undulatum*, *C. edule*); and tobacco (*Nicotianum tabacum*), named after the indigenous tobacco species (*N. attentuata*, *N. multivalvis*). Broad-leaved plantain (*Plantago major*), named *’laanaa łgunga*, “village skunk-cabbage”, in Haida (Skidegate dialect), and *g^w^íxsa k’ik’eʔuk^w^*, “looks like skunk-cabbage”, in Kwak’wala, serves as another example.

Other new species were named for their particular notable features, such as the blood-red color of beets or the prickliness of burdock. Examples include, for the former, the Tsilhqot’in name for beets (*Beta vulgaris*), *baxadadelnetezh* (lit. “blood runs out”) and the Stl’atl’imx name *tsíʔiʕ^w^’* (“bleed”), and for the latter, the Nuxalk name for burdock (*Arctium minus*), *mtm* (“sea urchins”). Still, other plants were named for particular associations, such as a number of other names for broad-leaved plantain that connect it with frogs: Ts’msyen (Sm’algyax): *naagənaw* (“frog’s dress”); W̱SÁNEĆ: *słéwən ʔə tsə sxəʔénəx^w^* (“mat/mattress/bed of the frog”); and Nlaka’pamux: *p’əp’ey’łeh-éytx^w^* (“frog-leaved”).

### 5.2. Misunderstandings Related to Land Occupancy and Traditional Plant Management

Despite the many benefits of the new plants imported into northwestern North America and into the lifeways of Indigenous Peoples, these same species, grown in European-style gardens and orchards, symbolized ignorance and disregard on the part of the newcomers. This was especially true of the Colonial officials who wanted to increase settlement in the region. They conveniently underrated the Indigenous Peoples’ use and occupancy of their lands, as well as their sophisticated and effective land and resource management practices which had been developing over millennia. These practices and associated knowledge—ways of planning and decision-making, intergenerational monitoring, and oversight—had been effective in maintaining Indigenous People’s overall health and well-being for countless generations, as described in other publications [2,42,43,44,45]. The lack of recognition of these practices by the newcomers, and the assumption that the Europeans were much more “civilized”, were used as justification for many actions and decisions that were harmful. These included the imposition of the residential schools, often run by racist church officials; the takeover of vast tracts of Indigenous lands; the destruction of fisheries, forests, prairies, and other natural areas through unsustainable harvesting practices; the draining of wetlands; and industrial-scale agriculture, all occurring alongside the introduction and adoption of the new species described here. New diseases like smallpox and tuberculosis were also taking a massive toll on the lives of the First Peoples. Even those newcomers who were well-meaning and sincerely attempting to improve the lives of the Indigenous People were still convinced that they needed to be converted to an agrarian lifestyle. Many considered it their God-given right and duty to “improve” the lives and cultures of the Indigenous Peoples. Self-interest, prejudice, ignorance, and misunderstanding enabled colonial and church officials, as well as the settlers, to readily overlook the social and ethnoecological systems already in place that were, in turn, maintaining and supporting the existing plants [15,46,47].

The First Peoples themselves were often conscripted to transform their lands for agricultural production. In 1851, for example, James Douglas, Governor of the Colony of Vancouver Island, reported during the construction of Fort Victoria that “We have about 100 Indians employed in clearing the Brush and trees and bringing new land into cultivation”. Similar situations of First Peoples being hired as laborers to transform the landscape occurred in many places.

At least some First Nations people were resigned to the new lifestyle imposed by the newcomers. They saw it as a matter of survival. Secwepemc elder Dr. Mary Thomas recalled, “I often heard my mother talk about this, that it [clearing the land for agriculture around Salmon Arm] wasn’t their way of life, but they had no choice. They had to accept the way they were taught, how to survive, was to chop down all these trees and cultivate it into European way of living. I guess that’s where we began to lose a lot of the traditional foods” (pers. comm. to NT, 1995).

Kwakwaka’wakw hereditary Clan Chief Adam Dick, Kwaxistalla, and his community members at Gwayee Village, Kingcome Inlet, were employed to build dykes around their traditional wild root vegetable gardens on the tidal flats at the mouth of the Inlet so that the land could be converted to ranchland, with large numbers of grazing cattle and sheep. They were paid with butter and other goods. Then, when the dykes were built, the new owners of the land—the family of the man who was to be appointed “Indian Agent” in the region—proceeded to exclude the local Kwakwaka’wakw people, cutting down their native crabapple trees so that local people would not trespass on their property [42].

### 5.3. Dietary Change and Its Impacts

The new plant foods were just the beginning of dietary changes that accelerated over the following generations with cumulative impacts. Indigenous Peoples’ diets and lifestyles were changed in myriad ways, including through the residential schools, where students were not only fed inferior and often unhealthy foods, but were conscripted as laborers to look after the gardens and fruit trees being raised around many of the schools (Dr. Mary Thomas, pers. comm. to NT, 1995). The overall impact was a loss of access to healthy Indigenous food and higher use of unhealthy marketed and processed food. This dietary transformation impacted Indigenous Peoples worldwide, and has been termed the “nutrition transition” [48,49]. As a result, diseases like diabetes and obesity have further taken their toll.

### 5.4. Environmental Change and Loss

**Figure 9:**
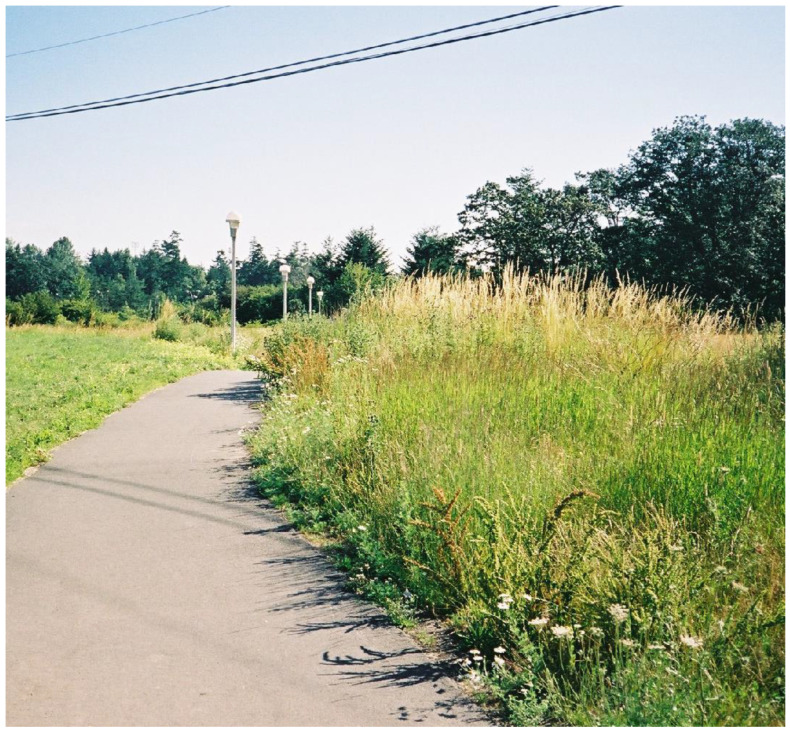
Weedy meadow on the grounds of University of Victoria, ca. 2015, in a place where there was formerly a dense camas meadow (*Camassia* spp.) within an oak prairie and many indigenous food species (N. Turner).

Meanwhile, the new plants, especially invasive weeds like couchgrass (*Elymus repens*), were taking their toll on the traditional Indigenous food plants and their habitats in many parts of the region. Meadows of camas (*Camassia* spp.), dense with nutritious bulbs and other food plants, were destroyed by grazing sheep and cattle and taken over by invasive grasses and broom (*Cytisus scoparius*) (Figure 9). Areas in Victoria that formerly produced immense patches of native springbank clover (*Trifolium wormskioldii*), with its edible rhizomes, were converted into lawns, planted with daffodils, or taken over by weedy grasses.

Everywhere, new habitats were produced where indigenous species grew interspersed with newly imported species, producing mixtures of flora which never before been encountered. Termed “novel ecosystems” [32,50], these became the “new normal” for vast areas of northwestern North America and many other parts of the world. Not only plants, but different species of mammals, birds, insects, fungi, and other life, have spread widely, leading to a homogenization of ecosystems and, with competition from more aggressive species, to an overall loss of indigenous biodiversity. In a cascading amalgamation of losses, the combination of declines in the original species and habitats from industrial-scale activities; loss of First Peoples’ access to their lands; suppression of the Potlatch and other ceremonial aspects of food as well as land use and care [2]; participation of First Peoples in the wage economy [51]; and multi-generational impacts of residential schools resulted in the loss or suppression of Indigenous knowledge of language and of care and use of the original foods. Fortunately, in many communities, some individuals were able to retain this knowledge through times of stress and change [52], and it is these individuals who have held enabled a resurgence of language and indigenous food use. At the same time, the imperative for conservation and restoration of native habitats and species, and for Indigenous Peoples to regain control and stewardship of their own territories, has received increasing support and attention in recent years [6,53,54].

### 5.5. The Specter of Climate Change

Despite the positive restoration and revitalization of Indigenous Peoples’ languages, cultures, and traditional foodways, including recent the revival and attention given to traditional plant foods, there is an ongoing worry that global climate change is having deep impacts on our ecosystems and on the well-being of all that live on earth, including humans. Indigenous Elders have observed changes in native plants, including a severe reduction in wild berry production, perhaps due to mis-timing in the blooming of the berry plants and the presence of insect pollinators [55]. Devastating fires and floods are increasing throughout the region, as they occur worldwide with further tolls on the indigenous plants, since once the soil is disturbed, there is a greater chance of invasive species like knapweed (*Centaurea diffusa*), cheatgrass (*Bromus tectorum*), foxglove (*Digitalis purpurea*), and tansy ragwort (*Jacobaea vulgaris*) taking hold. Ironically, non-native species like crested wheatgrass (*Agropyron cristatum*) have been purposefully planted over vast areas of burned and disturbed land in soil-conserving efforts.

Fortunately, all of the original food plant species of northwestern North America are still present in the region. Some of them have already served as sources of genetic stock for higher-yielding varieties and hybrids [33], and all of them still have the capacity to produce flavorful and nutritious food. There is also a real desire of Indigenous Peoples to restore these foods to their original statuses, both culturally and nutritionally [55,56]. Given their close relationships with humans going back to Pleistocene times, taken together with the new species that were brought into the region, they will hopefully continue in their life-giving roles as long as we humans are able to manage ourselves better, in order to mitigate and reverse the effects of climate change.

## 6. Conclusions

Many of the new plant foods and other plant species introduced to the region by European and other newcomers have found an important place in the diets and lifeways of the long-resident Indigenous Peoples. In numerous instances, however, and especially in more recent times, they have supplanted the original foods, medicines, and materials that had been key elements of Indigenous Peoples’ languages and cultures for millennia. This situation was exacerbated by other changes in people’s lives, as described previously (Section 5.4). This has resulted in other losses, especially in terms a people’s abilities to tend and manage their plant resources through the use of fire and other means [15,43,46]. Overall, this has impacted peoples’ health and well-being.

Great care will be needed if the important native food plants and other species used and tended by countless generations of Indigenous Peoples are to be maintained in the future. Not only are these species important for First Peoples’ cultures, languages, and overall well-being, but also for the myriad birds, mammals, insects, and other wildlife that depend on them for food and habitats. It will take concerted efforts in ethnoecological restoration, including immense reductions in fossil fuel use and habitat loss associated with oil and gas production, mining, and industrial agriculture to enable some of these original species to thrive. Elimination of many of the introduced species is neither possible nor necessarily desirable [57]; in fact, some might be considered as components of the natural process of learning about new techniques of food production and tending [58]. However, controlling their spread; restoring the habitats of the original species; and—for all of us, wherever we live in the world—recognizing the cultural and environmental values of native species within their original habitats, must be our ultimate goals.

## Figures and Tables

**Table 1:** Potato and other root crops Introduced to Northwestern North America by European and other newcomers in colonial times and adopted as food by Indigenous Peoples ([2], Supplement 1) (URL https://dspace.library.uvic.ca/handle/1828/5091 (accessed on 15 August 2023)).

Introduced Root Vegetables	Notes
*Allium cepa* (garden onion)	Planted at Nootka Sound by Spanish explorer Esté José Martínez, 1789 [16]; widely adopted and grown in gardens; named after wild onions (e.g., *Allium cernuum*) in some languages and for its odor in others (The Hesquiaht word for “body odour” translates to “smelling of onions”) [25]. Other names are derived from the English or French names; onions were named in over 10 languages of the region.
*Beta vulgaris* (beets)	Planted at Nootka Sound in 1792 [16]; introduced by early traders to NW N America in the 1800s; widely adopted and grown in gardens; often named after its red color (e.g., Syilx/Okanagan [7]); named in over 10 languages of the region.
*Brassica rapa* and related species (turnip, rutabaga)	Planted at Nootka Sound in 1789 [16]; widely adopted and grown in gardens; often named after English or Chinook Jargon names; Hesquiaht name translates as “looks like a skull” [25]; named in over 30 languages of the region.
*Daucus carota* (garden carrot)	Planted at Nootka Sound in 1792 [16]; introduced by early traders to NW N America in 1800s; named after wild relatives such as wild caraway (*Perideridia gairdneri*), or from English name; named in over 30 languages of the region
*Helianthus tuberosus* (Jerusalem artichoke)	Originally from northeastern N America; introduced locally in early times to a few lower BC mainland locales and grown in gardens; named in a few languages, in some after potato
*Pastinaca sativa* (garden parsnip)	Planted at Nootka Sound in 1792 [16]; not as widely known as turnips, potatoes, or carrots; named after wild relatives by some; named in at least 3 languages of the region
*Rhaphanus raphanistrum* subsp. *sativus* (radish)	Planted at Nootka Sound [16]; named in at least 1 language
*Solanum tuberosum* (potato)	Planted at Nootka sound [16]; introduced very early by traders to NW N America, possibly first from S. America, then from Europe where it had been imported from the New World in the 16th century and spread widely; named after English “good seed”, from French, or after wild tubers such as wapato (*Sagittaria latifolia*); widely adopted and grown [27]; named in all languages of the region.

**Table 3:** Green vegetables, legumes, and squashes introduced to Northwestern North America by European and other newcomers in colonial times and adopted as food by Indigenous Peoples.

Introduced Green Vegetables	Notes
*Apium sellowianum* (celery)	Planted by Spanish at Nootka Sound in 1792 [16] and adopted by some Indigenous groups; named by some after cow-parsnip (*Heracleum maximum*), which is similar in flavor
*Asparagus officinalis* (asparagus)	Introduced as a perennial garden green, mostly in the southern Interior, where it has “gone wild” in places; named in a few languages (e.g., Syilx/Okanagan)
*Brassica oleracea* (cabbage)	Planted at Nootka Sound by Spanish in 1789 [16]; adopted widely by Indigenous communities; commonly named after its English name or its big leaves; named in at least 12 languages
*Cucurbita maxima* (vegetable marrow, squash)	Widely grown in gardens; cooked in soups and stews; stored in raw form in the fall and winter; named for its shape, habit, or resemblance to melons; named in at least 11 languages
*Cucurbita pepo* (pumpkin)	Grown in some gardens; cooked in soups and desserts; named for its color and/or shape; named in at least 5 languages
*Lactuca sativa* (lettuce)	Planted at Nootka Sound by Spanish in 1789 [16] and grown in Indigenous gardens; often named for its large, green, edible leaves; sometimes has same name as spinach and other greens; named in at least 5 languages
*Lathyrus oleraceus* (syn. *Pisum sativum*) (peas)	Grown in gardens and peas; eaten fresh, as well as being acquired as marketed dried peas; named after the English name due to the rattling noise of the dried pods or after their shape (e.g., Nuu-chah-nulth term for peas in a pod: “inside a canoe”); named in at least 15 languages
*Phaseolus vulgaris* (beans)	Grown in gardens and eaten as green beans; dried beans are marketed for soup, etc.; named from English “beans” or resemblance to familiar objects (e.g., periwinkles in Nuu-chah-nulth; wood ticks in Ktunaxa); named in at least 8 languages
*Rheum rhabarbarum* (rhubarb)	Introduced and planted in many camps and settlements by miners and prospectors; readily adopted into Indigenous foodways and grown in gardens; named after native cow-parsnip (*Heracleum maximum*) (e.g., Okanagan/Hesquiaht) or rhubarb’s relative, western dock (*Rumex occidentalis*) (e.g., Haida); named in at least 12 languages

**Table 4:** Beverage and flavoring plants.

Introduced Beverage and Flavoring Plants	Notes
*Humulus lupulus* (common hops)	Many people earned a living picking hops in the Fraser and Cowichan Valleys and elsewhere; some started to grow hops as ornamental vines; hops are named in at least 10 languages, with terms generally based on the English name
*Mentha piperita* (peppermint)	Grows around many old village sites in the region; leaves are used for tea and flavoring; named in Haida for its good scent
*Mentha spicata* (spearmint)	Grows around many old village sites; leaves used for tea and flavoring; by some given the same as wild mint (*M. arvensis*), and by others named after hedge nettle (*Stachys chamissonis* var. *cooleyae*)
*Nepeta cataria* (catnip)	Adopted and grown by some peoples of the Interior, notably Syilx/Okanagan, who named it after its blue/green leaves or for its skunky odor; people have used it to make medicinal teas

## Data Availability

No new data were created or analyzed in this study. Data sharing is not applicable to this article.

## Appendix A

Species mentioned in the paper were characterized according to the patronymic, family, chorotype, and life form of each, based on Plants of the World Online (POWO) website: (https://powo.science.kew.org/ accessed 7–16 August 2023; Royal Botanical Gardens in Kew, London), and on the taxonomic backbone maintained by the International Plant Name Index (IPNI) (URL: https://bplant.org/website/17 (accessed on 16 August 2023)).

**Table A1:** Introduced plant species mentioned in this paper (by alphabetical order of botanical names).

Patronymic	Family	Climate/Original Geographical Distribution	Life Form
*Agropyron cristatum* (L.) Gaertn. (crested wheatgrass)	Poaceae	Temperate: Eurasia, N Africa	Perennial
*Allium cepa* L. (garden onion)	Amaryllidaceae	Temperate: Central Asia	Bulbous geophyte
*Ananas comosus* (L.) Merr. (pineapple)	Bromeliaceae	Tropical: Costa Rica, S Tropical America	Perennial
*Angelica archangelica* L. (European angelica)	Apiaceae	Temperate: Greenland, Europe, W Siberia	Temperate
*Apium sellowianum* H. Wolff (celery)	Apiaceae	Subtropical: Bolivia to Brazil, SW Argentina	Perennial
*Arctium minus* (Hill) Bernh. (burdock)	Asteraceae	Temperate: NW Africa, Europe, to W Siberia, Afghanistan	Biennial
*Asparagus officinalis* L. (asparagus)	Asparagaceae	Temperate: Europe to Mongolia	Perennial
*Avena sativa* L. (oats)	Poaceae	Temperate: W Asia	Annual
*Bambusa bambos* (L.) Voss. (bamboo)	Poaceae	Wet tropics: Indian Subcontinent to Indochina	Perennial
*Beta vulgaris* L. (beet)	Amaranthaceae	Temperate: W Europe, Mediterranean, India	Biennial or perennial
*Brassica oleracea* L. (cabbage)	Brassicaceae	Temperate: UK, Europe	Biennial or subshrub
*Brassica rapa* L. and related species (turnip, rutabaga)	Brassicaceae	Temperate: Mediterranean, Middle East, N Africa	Annual or biennial
*Bromus tectorum* L. (cheatgrass)	Poaceae	Temperate: Europe to Mongolia	Annual
*Camellia sinensis* (L.) Kuntze (tea)	Theaceae	Subtropical: E Asia to S China, N Indo-China	Shrub
*Capsicum annuum* L. (green pepper)	Solanaceae	Wet Tropics: Mexico to Guatemala	Annual or biennial
*Centaurea cyanus* L. (bachelor’s button)	Asteraceae	Temperate: C to E Mediterranean	Annual
*Centaurea diffusa* Lam. (knapweed)	Asteraceae	Temperate: Europe, SW Iberia, Caucasus	Biennial
*Chenopodium album* L. (lambsquarters)	Amaranthaceae	Temperate: Indian Subcontinent	Annual
*Cicer arietinum* L. (chickpea)	Fabaceae	Temperate: SE Turkey to Iran	Annual
*Cirsium arvense* (L.) Scop. (Canada thistle)	Asteraceae	Temperate: Eurasia, NW Africa	Perennial
*Cirsium vulgare* (Savi) Ten. (Scottish thistle)	Asteraceae	Temperate: Europe, Siberia, Arabian Peninsula, NW Africa	Biennial
*Citrullus lanatus* (Thunb.) Matsum. and Nakai (watermelon)	Cucurbitaceae	Dry tropical: E Sahara	Annual
*Citrus x sinensis* (L.) Osbeck (orange)	Rutaceae	Subtropical: S China	Tree or shrub
*Citrus x limon* (L.) Osbeck (lemon)	Rutaceae	Subtropical: hybrid Asia, India	Tree or shrub
*Coffea arabica* L. (coffee)	Rubiaceae	Dry tropical: Sudan, Ethiopea	Shrub, small tree
*Convolvulus arvensis* L. (field bindweed)	Convolvulaceae	Temperate, subtropical Old World	Perennial
*Cucumis melo* L. (cantaloupe)	Cucurbitaceae	Dry tropical: Ethiopia, S Africa, Middle East, India, Australia	Annual
*Cucurbita maxima* Duchesne (vegetable marrow, squash)	Cucurbitaceae	Subtropical: Bolivia to N Argentina	Annual
*Cucurbita pepo* L. (pumpkin)	Cucurbitaceae	Subtropical: cultigen, Mexico	Annual
*Cynara cardunculus* L. (globe artichoke)	Asteraceae	Temperate: Mediterranean	Perennial
*Cytisus scoparius* (L.) Link (common broom)	Fabaceae	Temperate: Europe	Shrub
*Daucus carota* L. (garden carrot)	Apiaceae	Temperate: Macronesia, NW Africa, Europe, S China	Biennial
*Digitalis purpurea* L. (foxglove)	Plantaginaceae	Temperate: W and SW Europe, Morocco	Biennial or perennial
*Elymus repens* (L.) Gould. (quackgrass)	Poaceae	Temperate: Eurasia, N Africa	Perennial
*Ficus carica* L. (fig)	Moraceae	Temperate: E Mediterranean to C Asia	Tree
*Fragaria* L. X (cultivated strawberries)	Rosaceae	Temperate: N hemisphere, C and S America, Hawaiian Islands	Perennial
*Galium aparine* L. (bedstraw)	Rubiaceae	Temperate: Macaronesia to Eurasia	Annual
*Helianthus tuberosus* L. (Jerusalem artichoke)	Asteraceae	Temperate: C and E Canada and USA	Tuberous geophyte
*Hordeum vulgare* L. (barley)	Poaceae	Temperate: E Mediterranean to C Asia and China	Annual
*Humulus lupulus* L. (hops)	Cannabaceae	Temperate: Europe to Siberia, N Iran, Morocco	Perennial
*Iris pseudacorus* L. (yellow iris)	Iridaceae	Temperate: Europe, Caucasus, Mediterranean to Iran	Rhizomatous geophyte
*Jacobaea vulgaris* (tansy ragwort)	Asteraceae	Temperate: Europe to Mongolia and Caucasus	Perennial
*Koenigia polystachya* (Wall. Ex Misn.) T.M. Schust. and Reveal (*syn. Persicaria wallichii* Greuter and Burdet) (Himalayan knotweed)	Polygonaceae	Temperate: Afghanistan to S C China	Perennial
*Lactuca sativa* L. (lettuce)	Asteraceae	Temperate: W Asia	Annual or biennial
*Lathyrus oleraceus* Lam. (syn. *Pisum sativum* L. (pea)	Fabaceae	Temperate: Mediterranean to Afghanistan	Annual or perennial
*Malus domestica* (Suckow) Borkh. (apple)	Rosaceae	Temperate: Afghanistan to C Asia	Tree
*Matricaria discoidea* DC (pineappleweed)	Asteraceae	Temperate: subarctic America	Annual
*Medicago sativa* L. (alfalfa)	Fabaceae	Temperate: Mediterranean to W Siberia and Iran	Annual or perennial
*Melilotus albus* Medik. (white sweet-clover)	Fabaceae	Temperate: Europe, China, N and S Africa	Annual or biennial
*Melilotus officinalis* (L.) Lam. (yellow sweet-clover)	Fabaceae	Temperate: Europe to W Himalyan, Arabian Peninsula	Annual or perennial
*Mentha x piperita* L. (peppermint)	Lamiaceae	Temperate: Europe, C Asia	Perennial
*Mentha spicata* L. (spearmint)	Lamiaceae	Temperate: Europe to China	Perennial
*Mentha* spp. (mints)	Lamiaceae	Temperate:	Perennial
*Musa x paradisiaca* L. (banana)	Musaceae	Wet tropical: Malesia	Herbaceous tree
*Nasturtium officinale* W. T. Aiton (watercress)	Brassicaceae	Temperate: Europe to C Asia, Arabian Peninsula	Perennial or helophyte
*Nepeta cataria* L. (catnip)	Lamiaceae	Temperate: S Europe to Japan	Perennial
*Nicotiana tabacum* L. (tobacco)	Solanaceae	Temperate: Bolivia	Annual or perennial
*Oryza sativa* L. (rice)	Poaceae	Temperate: cultigen from China	Annual or helophyte
*Pastinaca sativa* L. (garden parsnip)	Apiaceae	Temperate: Europe to C Siberia and Lebanon	Biennial
*Phalaris arundinacea* L. (reed canary grass, or ribbon grass)	Poaceae	Temperate: temperate and subtropical Northern Hemisphere to Tropical mountains	Perennial or rhizomatous geophyte
*Phaseolus vulgaris* L. (beans)	Fabaceae	Dry tropical: Mexico and C America	Annual
*Phleum pratense* L. (timothy grass)	Poaceae	Temperate: Azores, Morocco, Europe to Siberia and W Himalaya	Perennial
*Plantago major* (broad-leaved plantain)	Plantaginaceae	Temperate: Eurasia, Arabian Peninsula, Macaronesia, N and S Africa	Annual or perennial
*Prunus domestica* L. (garden plum)	Rosaceae	Temperate: Transcaucasus to N Iran	Tree
*Prunus avium* (L.) L. (sweet cherry)	Rosaceae	Temperate: Europe to Afghanistan, N. Africa	Tree
*Prunus cerasus* L. (sour cherry)	Rosaceae	Temperate: Caucasus	Tree
*Prunus persica* (L.) Batsch (peach)	Rosaceae	Temperate: N C China	Tree
*Pyrus communis* L. (pear)	Rosaceae	Temperate: Europe to N Iraq	Tree
*Ranunculus acris* L. (meadow buttercup) and other *Ranunculus* spp.	Ranunculaceae	Temperate: Greenland, Europe to E. Russia	Perennial
*Raphia farinifera* (Gaertn.) Hyl. (Raphia palm)	Arecaceae	Wet tropical: Tropical Africa, Comoros, N and E Madagascar	Shrub
*Reynoutria japonica* Houtt. (Japanese knotweed)	Polygonaceae	Temperate: Russian Far East to China, E Asia	Perennial or rhizomatous geophyte
*Raphanus raphanistrum* subsp. *sativus* (L.) Domin (radish)	Brassicaceae	Temperate: Mediterranean	Annual or biennial
*Rheum rhabarbarum* L./*hybridum* (rhubarb)	Polygonaceae	Temperate: S Siberia to N and C China	Perennial
*Ribes nigrum* L. (black garden currant)	Grossulariaceae	Temperate: Europe to Russia Far E and W Himalaya	Shrub
*Ribes rubrum* L. (red garden currant)	Grossulariaceae	Temperate: W. Europe	Shrub
*Ribes uva-crispa* L. (gooseberries)	Grossulariaceae	Temperate: Europe, NW Africa, Turkey, Iran	Shrub
*Rosa* spp. (ornamental roses)	Rosaceae	Temperate: Temperate and Subtropical N Hemisphere	Shrubs
*Rubus allegheniensis* Porter (Allegheny blackberry)	Rosaceae	Temperate: E N America	Shrub
*Rubus armeniacus* Focke (Himalayan blackberry)	Rosaceae	Temperate: Transcaucasus to N Iran	Shrub
*Rubus nemoralis* P.J. Mull. (cutleaf blackberry)	Rosaceae	Temperate: N and C Europe	Shrub
*Rubus* L. hybrids (loganberry, boysenberry and related hybrids of blackberries and raspberries)	Rosaceae	Temperate: widespread, Europe, N America, temperate, subtropical, tropical mountains	Shrubs & vines
*Rubus idaeus* L. (raspberry)	Rosaceae	Temperate: N hemisphere	Shrub
*Rumex acetosella* L. (sourgrass or sheep sorrel)	Polygonaceae	Temperate: Eurasia	Perennial
*Rumex crispus* L. and other *Rumex* spp. (curly dock and related dock species)	Polygonaceae	Temperate: Macaronesia, N Africa, temperate Eurasia	Annual or perennial
*Saccharum officinarum* L. (sugar cane)	Poaceae	Dry Tropical: New Guinea	Perennial or rhizomatous geophyte
*Syringa vulgaris* L. (lilac)	Oleaceae	Temperate: C Albania to N C Romania	Shrub or tree
*Solanum lycopersicum* L. (tomato)	Solanaceae	Wet tropical: Peru	Subshrub
*Solanum melongena* L. (eggplant)	Solanaceae	Tropical, subtropical: W Indian Ocean, tropical and subtropical Asia	Shrub
*Solanum tuberosum* L. (potato)	Solanaceae	Subtropical: W and S S America and NW Venezuela	Tuberous geophyte
*Taraxacum officinale* F. H. Wigg (common dandelion)	Asteraceae	Temperate: Macaronesia, Europe to Siberia, NW Africa	Perennial
*Tragopogon pratensis* L. (salsify or goatsbeard)	Asteraceae	Temperate: Europe to Central Asia, Turkey	Biennial
*Trifolium pratense* L. (red clover)	Fabaceae	Temperate: Macaronesia, NW Africa, Europe to Mongolia & Himalaya	Perennial
*Trifolium repens* L. and other *Trifolium* spp. (white-flowered clovers)	Fabaceae	Temperate: Europe, NW Africa to Mongolia	Perennial
*Triticum aestivum* L. (wheat)	Poaceae	Temperate: Transcaucasus, Middle East to NW India	Annual or biennial
*Vaccinium corymbosum* L. (highbush blueberry)	Ericaceae	Temperate: E N America	Shrub
*Vaccinium* spp. (other cultivated and hybrid blueberry vars.)	Ericaceae	Temperate: Cosmopolitan	Shrubs
*Verbascum thapsus* L. (common mullein)	Scrophulariaceae	Temperate: Azores, Europe to Siberia, Himalaya	Biennial
*Viola tricolor* L. and hybrids (pansy)	Violaceae	Temperate: Europe to W Siberia and NW Iran	Annual or subshrub
*Vitis vinifera* L. (grape)	Vitaceae	Temperate: S Central and SE Europe to C Asia and N Iran	Woody vine
*Zea mays* L. (corn, maize)	Poaceae	Dry tropical: C and SW Mexico to W Guatemala	Annual
*Zizania aquatica* L. var. *interior* Fasset (wildrice)	Poaceae	Temperate: C and E Canada to C and NE USA	Annual

**Table A2:** Plant species mentioned in this paper native to NW North America (by alphabetical order of botanical names).

Patronymic	Family	Climate/Original Geographical Distribution	Life Form
*Acorus calamus* L. var. *americanus* Raf. (sweetflag)	Acoraceae	Temperate: C Siberia to Mongolia, Subarctic America to N & E USA	Perennial or rhizomatous geophyte
*Amelanchier alnifolia* (Nutt.) Nutt. ex M. Roem. (saskatoonberry)	Rosaceae	Temperate: Subarctic America to W and C USA	Shrub or tree
*Anthoxanthum nitens (Weber) Y. Schouten and Veldkamp [syn.* *Hierochloë hirta (Schrank) Borbás] (*sweetgrass)	Poaceae	Subarctic and Temperate: N and C N America	Perennial or rhizomatous geophyte
*Arctostaphylos uva-ursi* (L.) Spreng. (bearberry or kinnikinnick)	Ericaceae	Temperate: Subarctic America to NW and C USA	Subshrub
*Asarum caudatum* Lindl. (wild ginger)	Aristolochiaceae	Temperate: W N America	Perennial or rhizomatous geophyte
*Camassia leichtlinii* (Baker) S. Wats. (great camas)	Asparagaceae (formerly Liliaceae)	Temperate: W N America (S BC to C CA)	Bulbous geophyte
*Camassia quamash* (Pursh) Greene (common camas)	Asparagaceae (formerly Liliaceae)	Temperate: W Canada to W USA	Bulbous geophyte
*Cirsium edule* Nutt. (edible thistle)	Asteraceae	Temperate: W N America	Perennial
*Cirsium undulatum* Spreng. (wavy-leaved thistle)	Asteraceae	Temperate: W and C Canada to N Mexico	Perennial
*Corylus cornuta* Marshall (hazelnut)	Betulaceae	Temperate: W N America	Shrub
*Frangula purshiana* (DC) A. Gray ex J.G. Cooper (cascara)	Rhamnaceae	Temperate: W Canada to Mexico	Tree or shrub
*Marah oregana* (Torr. and A. Gray) Howell (manroot)	Cucurbitaceae	Temperate: SW Canada to N California	Climbing geophyte
*Mentha arvensis* L. (wild mint)	Lamiaceae	Temperate: Circumboreal; N America, Europe to Kamchatka and Nepal	Perennial
*Nicotiana attenuata* Torr. Ex S. Watson, *N. quadrivalvis* Pursh var. *quadrivalvis* (native tobaccos)	Solanaceae	Temperate: *N. attenuata*—W Canada to NW Mexico; *N. quadrivalvis—*Oregon	Annuals
*Oplopanax horridus* (Sm.) Miq. (devil’s-club)	Araliaceae	Temperate: W N America, Ontario	Shrub
*Pseudoroegneria spicata* (Pursh) Á. Löve (perennial bunchgrass)	Poaceae	Temperate: W and C N America to N Mexico	Perennial
*Rhododendron groenlandicum* (Oeder) Kron and Judd (Labrador tea)	Ericaceae	Temperate: Subarctic America to N USA	Shrub or subshrub
*Ribes hudsonianum* Richardson (black currant)	Grossulariaceae	Temperate: Subarctic America to N and W USA	Shrub
*Rumex occidentalis* S. Wats. (native western dock)	Polygonaceae	Temperate: Subarctic N America to N, W and C USA	Perennial
*Sagittaria latifolia* Willd. (wapato)	Alismataceae	Temperate: Canada to C & E USA, Cuba, California to W S America	Tuberous geophyte
*Shepherdia canadensis* (L.) Nutt. (soapberry)	Elaeagnaceae	Temperate: Subarctic N America to W Central and N USA	Shrub
*Stachys chamissonis* var. *cooleyae* (A. Heller) G.A. Mulligan and D.B. Munro (hedge nettle)	Lamiaceae	Temperate: W Canada and W USA	Perennial
*Trifolium wormskioldii* Lehm. (springbank clover)	Fabaceae	Temperate: W and C N America to Mexico	Perennial
*Urtica dioica* L. (stinging nettle)	Urticaceae	Temperate: widespread Europe to Siberia and W China (also N N America)	Perennial to rhizomatous geophyte
*Vaccinium membranaceum* Dougl. Ex Torr. (black huckleberry)	Ericaceae	Subalpine/subarctic: W & E Canada to N and W USA	Shrub or subshrub
*Vaccinium myrtilloides* Michx. (Canadian blueberry)	Ericaceae	Temperate: Subarctic America to N USA	Shrub or subshrub
*Vaccinium oxycoccos* (cranberry)	Ericaceae	Temperate: Subarctic and temperate N Hemisphere	Subshrub
*Veratrum viride* Ait. (false hellebore)	Melanthiaceae	Temperate: W N America from Alaska to USA	Perennial or rhizomatous geophyte
*Viburnum edule* (Michx.) Raf. (highbush cranberry, or mooseberry)	Viburnaceae	Temperate: N Russian Far East, Subarctic to W N America to N USA	Shrub
*Xerophyllum tenax* (Pursh) Nutt. (beargrass)	Melanthiaceae	Temperate: W Canada to N California	Perennial

## Appendix B

**Table A3:** Names of Indigenous Peoples of Northwestern North America and their territories, shown by number in the Figure 2 map.

Language	Family	Cultural Area	Key on Map
Tlingit	Na-Dene	Northwest Coast	1
Haida (Massett, Alaska)	Haida	Northwest Coast	2
Haida (Skidegate)	Haida	Northwest Coast	3
Tanaina/Dena’ina (U, Iliamna and Inland dialects)	Na-Dene		4
Ahtna	Na-Dene	Sub-boreal	Not shown (Copper R valley, AK)
N. Tutchone	Na-Dene	Sub-boreal	5
S. Tutchone/Han			5a
Tahltan	Na-Dene	Sub-boreal	6
Gwich’in	Na-Dene	Sub-boreal	7
Slave	Na-Dene	Sub-boreal	7b
Beaver (Dunneza)	Na-Dene	Sub-boreal	8
Sekani	Na-Dene	Sub-boreal	9
Kaska (Liard)	Na-Dene	Sub-boreal	10
Witsuwet’in (Babine)	Na-Dene	Sub-boreal	11
Dakelh/Carrier (Stuart/Trembleur Lake)	Na-Dene	Sub-boreal	12
Dakelh/Carrier (Saik’uz)	Na-Dene	Sub-boreal	12
Dakelh/Carrier (Ulkatcho)	Na-Dene	Sub-boreal	12
Tsilhqut’in	Na-Dene	Sub-boreal	13
Nicola	Na-Dene	Plateau	14
Nisga’a	Tsimshianic	Northwest Coast	15
Gitxsan	Tsimshianic	Northwest Coast	16
Tsimshian (Sm’algyax)	Tsimshianic	Northwest Coast	17
Kitasoo (Haihais, Sgűűsx, Southern Tsimshian)	Tsimshianic	Northwest Coast	18
Haisla	Wakashan	Northwest Coast	19
Hanaksiala	Wakashan	Northwest Coast	20
Heiltsuk (Bella Bella)	Wakashan	Northwest Coast	21
Oweekeeno (Oowekyala)	Wakashan	Northwest Coast	22
Kwakwaka’wakw (Kwakiutl; speaking Kwak’wala and several other dialects)	Wakashan	Northwest Coast	23
Nuu-chah-nulth (Hesquiaht and many other dialects) (formerly, Nootka or Nootkans)	Wakashan	Northwest Coast	24
Ditidaht (Nitinaht) (sometimes included in Nuu-chah-nulth/Nootkans)	Wakashan	Northwest Coast	25
Makah	Wakashan	Northwest Coast	26
Nuxalk (Bella Coola)	Salishan	Northwest Coast	27
Tla A’min (Sliammon), Comox	Salishan	Northwest Coast	28
Sechelt	Salishan	Northwest Coast	29
Squamish (Skxwúmish)	Salishan	Northwest Coast	30
Straits Salish (northern: Saanich and other dialects)	Salishan	Northwest Coast	31
Klallam (Clallam)	Salishan	Northwest Coast	32
Samish	Salishan	Northwest Coast	33
Halkomelem, Halq’eméylem, Hul’qumi’num, Quw’utsun’ (Cowichan, Vancouver Island)	Salishan	Northwest Coast	34a Qualicum 34b Snuneymuxw 34c Quw’utsun’ 34d Esquimalt, Songhees, Saanich
Halkomelem, Upriver (Stó:lo) and Downriver (Musqueam)	Salishan	Northwest Coast	35
Lushootseed	Salishan	Northwest Coast	36
Twana	Salishan	Northwest Coast	37
Nooksack	Salishan	Northwest Coast	38
Tillamook	Salishan	Northwest Coast	39
Lower Chehalis	Salishan	Northwest Coast	40
Upper Chehalis	Salishan	Northwest Coast	41
Quinault	Salishan	Northwest Coast	42
Stl’atl’imx (Lil’wat, Pemberton)	Salishan	Interior Plateau	43
Stl’atl’imx (Fraser River)	Salishan	Interior Plateau	44
Nlaka’pmx	Salishan	Interior Plateau	45
Secwepemc (Fraser River) (W, E)	Salishan	Interior Plateau	46
Okanagan (Okanagan-Colville)	Salishan	Interior Plateau	47
Sinixt (Lakes)	Salishan	Interior Plateau	48
Columbian (Columbian-Wenachee; Middle Columbia River)	Salishan	Interior Plateau	49
Snchítsu’umshtsn (Coeur d’Alene)	Salishan	Interior Plateau	50
Spokan/Kalispel	Salishan	Interior Plateau	51
Selish (Flathead and Pend d’Oreille)	Salishan	Interior Plateau	52
Upper Cowlitz (Western Columbia River; Northwest Sahaptin)	Sahaptian (Penutian)	Interior Plateau	53
Sahaptin (including Yakima and neighbouring groups)	Sahaptian (Penutian)	Interior Plateau	54
Nez Perce	Sahaptian (Penutian)	Interior Plateau	55
Ktunaxa (Kootenai)	Kutenai	Interior Plateau	56
Quileute	Chemakuan	Northwest Coast	57
Wasco/Wishram	Chinookan	Interior Plateau	58
Eyak	Na-Dené	Northwest Coast	59
Tagish	Athapaskan	Western Subarctic	60
Pentlatch	Salishan	Northwest Coast	61
Nicola Athabaskan	Athapaskan	Interior Plateau	62
Chemakum	Chimakuan	Lower Columbia	63
Clatskanie, Tlatskanai	Athapaskan	Lower Columbia	63b
Tillamook, Aslean	Salishan	Northwest Coast	64
Siusiawan	Penutian?	Northwest Coast	65
Coosas	Coosan	Northwest Coast	66
Kalapuya (Willamette Valley)	Sahaptian (Penutian)	Northwest Coast	67
Cayuse (Umatilla and Walla Walla), Molala	Penutian	Plateau	68
Klamath and Modoc	Penutian	Plateau	69

## References

[1] Hofman C.A., Torben C.R. (2018). Ancient biological invasions and island ecosystems: Tracking translocations of wild plants and animals. J. Arch. Res..

[2] Turner N.J. (2014). Ancient Pathways, Ancestral Knowledge: Ethnobotany and Ecological Wisdom of Indigenous Peoples of Northwestern North America.

[3] Turner N.J., von Aderkas P. (2012). Sustained by First Nations: European Newcomers’ Use of Indigenous Plant Foods in Temperate North America. Acta Soc. Bot. Pol..

[4] Turner N.J., Thompson L.S., Thompson M.T., York A.Z. (1990). Thompson Ethnobotany. Knowledge and Usage of Plants by the Thompson Indians of British Columbia.

[5] Turner N.J. (2018). Learning new medicines: Exchanging medicinal plant knowledge amongst northwestern North American Indigenous and settler communities. Med. Nei Secoli.

[6] Turner N.J. (2020). Plants, People, and Places: The Roles of Ethnobotany and Ethnoecology in Indigenous Peoples’ Land Rights in Canada and Beyond.

[7] Turner N.J., Bouchard R., Kennedy D.I.D. (1981). Ethnobotany of the Okanagan-Colville Indians of British Columbia and Washington.

[8] Turner N.J., Thomas J., Carlson B.F., Ogilvie R.T. (1983). Ethnobotany of the Nitinaht Indians of Vancouver Island.

[9] (2022). TCPS 2. Tri-Council Policy Statement: Ethical Conduct for Research Involving Humans. https://ethics.gc.ca/eng/policy-politique_tcps2-eptc2_2022.html.

[10] Reimer R. (2015). Reassessing the role of Mount Edziza obsidian in northwestern North America. J. Arch. Sci. Rep..

[11] Teit J.A. (1909). The Shuswap.

[12] Thompson L., Doonan R.C.P. (2019). Copper on the Northwest Coast, a biographical approach. J. Arch. Sci. Rep..

[13] Turner N.J., Loewen D.C. (1998). The original “Free Trade”: Exchange of botanical products and associated plant knowledge in Northwestern North America. Anthropologica.

[14] Turner N.J., Taylor R.L. (1972). A review of the Northwest Coast tobacco mystery. Syesis.

[15] Turner N.J., Armstrong C.G., Lepofsky D. (2021). Adopting a root. Documenting ecological and cultural signatures of plant translocations in Northwestern North America. Am. Anthropol..

[16] Justice C.L. (2000). Mr. Menzies’ Garden Legacy: Plant Collecting on the Northwest Coast.

[17] Mackie R.S. (1984). Colonial Land, Indian Labour and Company Capital: The Economy of Vancouver Island, 1849–1858. Master’s Thesis.

[18] Turner N.J., Brown C.H., Gerdts D.B., Matthewson L. (2004). Grass, hay, and weedy growth: Utility and semantics of Interior Salish botanical terms. Studies in Salish Linguistics in Honor of M. Dale Kinkade.

[19] Black M.J., Ford R.I. (1994). Plant dispersal by Native North Americans in the Canadian Subarctic. The Nature and Status of Ethnobotany.

[20] Gilmore M.R. (1931). Dispersal by Indians a Factor in the Extension of Discontinuous Distribution of Certain Species of Native Plants. Pap. Mich. Acad. Sci. Arts Lett..

[21] Turner N.J. (2021). Plants of Haida Gwaii. Xaadaa Gwaay guud gina k’aws (Skidegate), Xaadaa Gwaayee guu giin k’aws (Massett).

[22] Turner N.J. (2018). Sweetgrass. Can. Encycl..

[23] Moerman D. (2003). Native American Ethnobotany. A Database of Foods, Drugs, Dyes and Fibers of Native American Peoples, Derived from Plants.

[24] Klinkenberg B. (2020). Acorus Americanus. E-Flora BC: Electronic Atlas of the Plants of British Columbia [eflora.bc.ca].

[25] Turner N.J., Efrat B.S. (1982). Ethnobotany of the Hesquiat Indians of Vancouver Island.

[26] Turner N.J., Hebda R.J. (2012). Saanich Ethnobotany: Culturally Important Plants of the WSÁNEC’ People.

[27] Suttles W. (1951). The early diffusion of the potato among the Coast Salish. SW J. Anthropol..

[28] Brown C.R. The Potato of the Makah Nation. The NSF Potato Genome Project: Outreach. http://www.potatogenome.org/nsf3/outreach/makah/the_makah_potato.php.

[29] Wenstob S. (2011). The profusion of potatoes in Pre-Colonial British Columbia. Platform.

[30] Zhang L., Brown C.R., Culley D., Baker B., Kunibe E., Denney H., Smith C., Ward N., Beavert T., Coburn J. (2010). Inferred origin of several Native American potatoes from the Pacific Northwest and Southeast Alaska using SSR markers. Euphytica.

[31] Work J., Dee H.D. (1945). The Journal of John Work, January to October, 1835.

[32] Gomes T.C. (2013). Novel ecosystems in the restoration of cultural landscapes of *Tl’chés*, West Chatham Island, British Columbia, Canada. Ecol. Process.

[33] Nabhan G. (2006). Renewing Salmon Nation’s Food Traditions.

[34] McDonald J.A., Deur D., Turner N.J. (2005). Cultivating in the Northwest: Early accounts of Tsimshian horticulture. “Keeping It Living”: Traditions of Plant Use and Cultivation on the Northwest Coast of North America.

[35] Armstrong C.G. (2022). Silm Da’axk: Historical Ecology and Ethnobotany in Gitselasu Lahkhyuup.

[36] Armstrong C.G., Miller J., McAlvay A., Ritchie P., Lepofsky D. (2021). Historical Indigenous land-use explains plant functional trait diversity. Ecol. Soc..

[37] Lepofsky D., Letham B., Ritchie M., Armstrong C.G., Fitzpatrick S., Erlandson J. (2023). Placemaking on the Northwest Coast. The Oxford Handbook of Island and Coastal Archaeology.

[38] Turner N.J. (1973). Ethnobotany of the Bella Coola Indians of British Columbia. Syesis.

[39] E-Flora B.C., Meidinger D., Lee T., Douglas G.W., Britton G., MacKenzie W., Qian H. (2012). Invasive, Noxious and Problem Plants of British Columbia (Checklist).

[40] Teit J.A., Boas F. (1930). The Salishan Tribes of the Western Plateaus.

[41] Berlin B. (1992). Ethnobiological Classification: Principles of Categorization of Plant and Animals in Traditional Societies.

[42] Turner N.J., Turner K.L. (2008). “Where our women used to get the food”: Cumulative effects and loss of ethnobotanical knowledge and practice; case studies from coastal British Columbia. Botany.

[43] Turner N.J., Berkes F. (2006). Developing Resource Management and Conservation. Hum. Ecol..

[44] Turner N.J., Gregory R., Brooks C., Failing L., Satterfield T. (2008). From Invisibility to Transparency: Identifying the Implications (of invisible losses to First Nations communities). Ecol. Soc..

[45] Turner N.J., Berkes F., Stephenson J., Dick J. (2013). Blundering intruders: Multi-scale impacts on Indigenous food systems. Hum. Ecol..

[46] Deur D., Turner N.J. (2005). “Keeping It Living”: Traditions of Plant Use and Cultivation on the Northwest Coast of North America.

[47] Corntassel J., Bryce C. (2012). Practicing Sustainable Self-Determination: Indigenous Approaches to Cultural Revitalization and Restoration. Brown J. World Aff..

[48] Kuhnlein H.V., Erasmus B., Spigelski D. (2009). Indigenous Peoples’ Food Systems. The Many Dimensions of Culture, Diversity and Environment for Nutrition and Health.

[49] Kuhnlein H.V., Erasmus B., Spigelski D., Burlingame B. (2013). Indigenous Peoples’ Food Systems & Well-Being: Interventions & Policies for Healthy Communities.

[50] Hobbs R.J., Higgs E., Hall C.M. (2013). Novel Ecosystems: Intervening in the New Ecological World Order.

[51] Lutz J.S. (2008). Makuk: A New History of Aboriginal-White Relations.

[52] Luschiim A., Charlie Turner N.J. (2021). Luschiim’s Plants: A Hul’q’umi’num’ (Cowichan) Ethnobotany.

[53] Senos R., Lake F., Turner N.J., Martinez D., Apostol D. (2006). Traditional Ecological Knowledge and Restoration Practice in the Pacific Northwest. Encyclopedia for Restoration of Pacific Northwest Ecosystems.

[54] Spalding P.R. (2022). Decolonizing Landscapes: Applications of Ethnobotanical Research in Defining Aboriginal Rights and Re-Affirming Indigenous Laws in T’sou-ke Territory, Vancouver Island and Beyond. Ph.D. Thesis.

[55] Turner N.J., Clifton H. (2009). “It’s so different today.” Climate Change and Indigenous Lifeways in British Columbia, Canada. Glob. Environ. Change.

[56] Joseph L., Turner N.J. (2020). “The Old Foods are the New Foods!”: Reviving Indigenous foods in Northwestern North America. Spec. Coll. “Traditional Food Knowledge: New Wine into Old Wineskins?”. Front. Sustain. Food Syst..

[57] Shackleford N., Hobbs R.J., Heller N.E., Hallett L.M., Seastedt T.R. (2013). Finding a middle-ground; The Native/non-native debate. Biol. Conserv..

[58] Ngapo T.M., Bilodeau P., Arcand Y., Charles M.T., Diederichsen A., Germain I., Liu Q., MacKinnon S., Messiga A.J., Modor M. (2021). Historical Indigenous food preparation using produce of the three sisters intercropping system. Foods.

